# The Role of Psychedelics in Contemporary Psychological and Interdisciplinary Inquiry

**DOI:** 10.3390/jpm15100450

**Published:** 2025-09-28

**Authors:** Kerem Kemal Soylemez, Emma Marie de Boo, Aysil Susuzlu, Joanne Lusher

**Affiliations:** School of Psychology, Regent’s University London, London NW1 4NS, UK

**Keywords:** psychedelics, interdisciplinary, anthropology, philosophy, palliative care

## Abstract

Psychedelic compounds are gaining renewed attention across disciplines for their profound psychological and neurobiological effects. Emerging research highlights their efficacy in treating mood disorders, PTSD, and addiction by enhancing neuroplasticity and disrupting maladaptive cognitive patterns. From a psychological standpoint, psychedelics facilitate introspection, emotional processing, and therapeutic breakthroughs. Neuroscientific findings reveal altered brain network dynamics, while anthropological and philosophical perspectives contextualize their cultural and existential significance. In medicine, they offer novel interventions for chronic pain and palliative care. The present review article underscores the need for rigorous, ethically grounded research to explore psychedelics’ potential in reshaping mental health paradigms and cognitive science from a multidisciplinary perspective.

## 1. Introduction: The Psychedelic Renaissance

In recent decades, the field of psychedelic research has experienced a resurgence, often referred to as the “psychedelic renaissance”. After decades of prohibition and stigmatization, substances such as psilocybin, LSD, MDMA, and ayahuasca are once again being explored for their therapeutic and scientific potential [1]. This renewed interest is grounded in both historical context and contemporary necessity. During the mid-20th century, psychedelics’ potential in treating mental health conditions was investigated by researchers [2]. However, by the late 1960s, political and cultural backlash led to their criminalization and the suspension of nearly all clinical research. For over three decades, scientific engagement with these substances was minimal, with exceptions largely confined to anthropological or countercultural discourse [3].

The contemporary revival began gaining momentum in the early 2000s, driven by advances in neuroimaging technologies, shifting societal attitudes toward mental health, and an increasing body of anecdotal and historical evidence supporting the therapeutic use of psychedelics [4]. Institutions such as Johns Hopkins University, Imperial College London, and the Multidisciplinary Association for Psychedelic Studies (MAPS) have played central roles in legitimizing and advancing psychedelic science. As mental health crises escalate globally, with depression, anxiety, post-traumatic stress disorder (PTSD), and substance use disorders affecting millions, conventional pharmacotherapies often fall short in providing sustained relief [5]. Psychedelics offer a fresh perspective by targeting not only biochemical imbalances but also deeply ingrained cognitive and emotional patterns [6].

The renaissance is further fueled by a multidisciplinary approach. Neuroscientists investigate the effects of psychedelics on brain networks and neuroplasticity [7]. Psychologists explore the substances’ capacity to facilitate emotional processing and self-awareness [8]. Anthropologists and historians contextualize traditional uses in Indigenous rituals, offering insights into long-standing cultural practices [9]. Philosophers and ethicists examine the existential dimensions of psychedelic experiences, questioning notions of consciousness, identity, and healing [10]. This integrative perspective has redefined psychedelics not as mere recreational tools or dangerous substances but as potent instruments for inquiry and intervention.

Moreover, the technological and cultural landscapes of the 21st century have created a fertile ground for the revival of psychedelic science. Digital platforms and social media have amplified anecdotal reports and facilitated community-building among advocates, therapists, and researchers. At the same time, mental health professionals increasingly recognize the limitations of current treatments, particularly in resistant cases. This confluence of scientific innovation and cultural openness has established psychedelics as a promising frontier in both medicine and consciousness studies.

Nevertheless, the field remains in a formative stage, necessitating rigorous, ethically grounded research to confirm early findings and guide safe, effective applications. Regulatory frameworks are evolving, with some regions decriminalizing or legalizing psychedelic-assisted therapy [11], while others maintain strict prohibitions. The psychedelic renaissance is not only a scientific and medical movement but also a cultural and philosophical shift, one that reimagines the boundaries of mental health care and human experience.

## 2. Neurobiological Mechanisms of Psychedelic Action

Understanding the neurobiological mechanisms of psychedelics is essential for elucidating their therapeutic potential. Psychedelic compounds, particularly classic serotonergic psychedelics such as psilocybin, LSD, and DMT, primarily act as agonists at the 5-HT2A receptor, a subtype of the serotonin receptor system [12]. Husain suggests that this interaction triggers a series of changes that alter typical neural activity and enhance communication between separate areas of the brain. One of the most consistently observed phenomena is the temporary downregulation of the default mode network (DMN), a brain system associated with self-referential thought, rumination, and ego maintenance [13]. Hyperactivity within the DMN has been linked to various psychopathologies, including depression and anxiety [14]. Psychedelic-induced suppression of the DMN is thought to facilitate ego dissolution and a subsequent reduction in rigid, maladaptive cognitive patterns [15].

Another key neurobiological effect of psychedelics is the enhancement of neuroplasticity [16]. Preclinical studies in animal models have demonstrated that psychedelics can promote the growth of dendritic spines, synaptogenesis, and increased expression of brain-derived neurotrophic factor (BDNF) [17]. These changes suggest that psychedelics not only alter brain activity acutely but also promote long-term restructuring of neural circuits, which may underpin their enduring therapeutic effects. Human neuroimaging studies using fMRI and PET scans have corroborated these findings, showing increased global connectivity and decreased modular segregation within the brain [18].

The neurochemical effects of psychedelics also extend beyond serotonin receptors. Compounds like MDMA primarily act on serotonin transporters, leading to a surge in extracellular serotonin, as well as effects on dopamine and norepinephrine systems [19]. This pharmacological profile accounts for MDMA’s empathogenic and prosocial properties, which are particularly useful in the treatment of PTSD and relational trauma [20]. Meanwhile, ketamine, a dissociative anesthetic with psychedelic-like properties, acts as an NMDA receptor antagonist and has been shown to induce rapid antidepressant effects through glutamatergic pathways and downstream mTOR signaling [21].

Importantly, these neurobiological changes do not occur in a vacuum. The phenomenology of the psychedelic experience, characterized by perceptual distortions, emotional breakthroughs, and mystical-type experiences, is deeply intertwined with its therapeutic outcomes [22]. Current theories propose that the brain under psychedelics enters a state of heightened entropy or “criticality”, enabling flexible cognitive and emotional reorganization [23]. This aligns with the Relaxed Beliefs Under Psychedelics model (REBUS), which remains a hypothesis and posits that psychedelics reduce the precision of high-level priors in the brain’s predictive coding hierarchy, allowing for novel interpretations and experiences [24].

Emerging research has begun to explore individual variability in response to psychedelics based on genetic, neurobiological, and psychological traits. For example, differences in 5-HT2A receptor density, baseline DMN activity, or levels of trait openness may influence the intensity and quality of psychedelic experiences [25]. This line of inquiry is critical for developing personalized therapeutic strategies that maximize benefits while minimizing risks.

Animal studies have also shed light on the molecular pathways activated by psychedelics, revealing interactions with inflammatory markers and epigenetic regulators [26]. These findings open new avenues for understanding how psychedelics might modulate not only neural circuitry but also systemic physiological processes [27]. For instance, some evidence suggests that psychedelics may reduce markers of neuroinflammation, potentially contributing to their antidepressant effects [28].

As research advances, understanding the complex neurobiological mechanisms of psychedelic action will be crucial for refining therapeutic protocols, predicting individual responses, and developing next-generation compounds with optimized efficacy and safety profiles. Integrating molecular, systems-level, and phenomenological data will be key to unlocking the full potential of these substances in modern medicine.

## 3. Psychological Effects and Therapeutic Insights

The psychological effects of psychedelics are central to their therapeutic potential. These substances induce profound alterations in cognition, emotion, and perception, which, when properly supported and integrated, can catalyze meaningful psychological change [29]. Key psychological phenomena associated with psychedelic experiences include introspection, emotional processing, ego dissolution, and the integration of traumatic memories. These effects are neither random nor merely hallucinatory; rather, they are shaped by the individual’s psychological landscape and the context in which the substance is consumed, commonly referred to as “set and setting” [30].

One of the most consistently reported outcomes of psychedelic experiences is heightened introspection [31]. Under the influence of psychedelics, individuals often access thoughts, memories, and emotions that are typically suppressed or inaccessible in ordinary states of consciousness. This intensified self-awareness allows for the reevaluation of long-held beliefs, unresolved conflicts, and subconscious motivations [32]. In therapeutic contexts, such introspective insight can lead to breakthroughs in understanding one’s behavior, identity, and life narrative.

Emotional processing is another core component of the psychedelic experience. Individuals frequently report encountering deep-seated emotions, such as grief, guilt, fear, or love, that had been repressed or insufficiently addressed [33]. Rather than bypassing these emotions, psychedelics often facilitate their full expression and resolution. This emotional catharsis is thought to play a significant role in the alleviation of symptoms in conditions like depression and PTSD, where emotional avoidance and dysregulation are prominent features [34].

A particularly important aspect of the psychedelic state is ego dissolution, which refers to a temporary loss of one’s usual sense of self or identity boundaries [35]. Ego dissolution can engender a feeling of unity with others, nature, or the cosmos, often described in spiritual or mystical terms. While potentially disorienting, this state is associated with increased openness, reduced narcissism, and greater cognitive flexibility in the long term. In clinical trials, the intensity of mystical-type experiences has been positively correlated with therapeutic outcomes, suggesting that ego transcendence may be a key mechanism of healing [36].

Trauma integration under psychedelics also warrants special attention [37]. Unlike traditional psychotherapies that may take months or years to reach the roots of traumatic memory [38], psychedelics often bring these memories into conscious awareness rapidly and vividly [39]. In a supported environment, individuals can reprocess traumatic experiences with a sense of safety and detachment, facilitating their resolution. MDMA-assisted psychotherapy, for instance, has shown significant efficacy in treating PTSD by allowing patients to revisit traumatic memories without being overwhelmed by fear or distress [40].

These therapeutic effects are not merely products of suggestion or placebo. Controlled clinical trials have demonstrated sustained symptom reduction in treatment-resistant depression, anxiety, and addiction following just one or two psychedelic-assisted sessions [41]. Mechanistically, the psychological impact is believed to result from the temporary destabilization of rigid cognitive and emotional patterns, creating a window of opportunity for reorganization and growth. This capacity for psychological “reset” distinguishes psychedelics from traditional pharmacotherapies, which typically require daily administration and primarily manage symptoms.

Integration, which is the process of making sense of and incorporating insights from the psychedelic experience, is essential for lasting therapeutic benefit [42]. Without integration, the profound realizations and emotional breakthroughs may fade or become confusing. Therapeutic models increasingly emphasize preparation and post-session integration as critical components of psychedelic therapy [43]. Integration may involve psychotherapy, journaling, mindfulness practices, or community support, all aimed at reinforcing positive changes and addressing any lingering challenges.

The psychological effects of psychedelics extend far beyond hallucinations or altered perceptions. These compounds have been shown to enable access to unconscious cognitive and emotional content, facilitate emotional release, and induce a temporary dissolution of ego boundaries—processes that may underpin significant and enduring therapeutic outcomes [44]. Such findings highlight the critical need for rigorously designed therapeutic frameworks that optimize clinical efficacy while mitigating potential risks. As research continues, the challenge will be to translate these complex psychological mechanisms into standardized, scalable, and ethically robust clinical practices.

## 4. Clinical Applications in Psychiatry and Mental Health

The therapeutic potential of psychedelics in psychiatry and mental health is becoming increasingly recognized as evidence mounts from both clinical trials and observational studies [45]. Substances such as psilocybin, MDMA, ketamine, and ayahuasca are being systematically studied for their efficacy in treating a broad range of psychiatric disorders, including major depressive disorder (MDD), post-traumatic stress disorder (PTSD), generalized anxiety disorder (GAD), and substance use disorders (SUDs) [46]. These applications mark a paradigm shift in psychiatric care, offering novel interventions for patients who are unresponsive to existing treatments or who experience significant side effects from conventional pharmacotherapies.

One of the most compelling areas of research involves the use of psilocybin for treatment-resistant depression (TRD) [47]. Multiple randomized controlled trials have demonstrated that a single or a few doses of psilocybin, administered in conjunction with psychotherapy, can produce rapid and sustained reductions in depressive symptoms [48]. Unlike standard antidepressants, which may take weeks to begin working and must be taken daily, psilocybin often exerts noticeable effects within hours, with therapeutic benefits lasting weeks or even months [49]. The subjective intensity of the psychedelic experience, including mystical-type encounters and emotional breakthroughs, has been positively correlated with clinical outcomes [50].

In the context of PTSD, MDMA-assisted psychotherapy has yielded particularly promising results. PTSD, a condition marked by intrusive memories, hyperarousal, and emotional numbing, is notoriously difficult to treat with existing pharmacological agents [51]. MDMA’s unique pharmacological profile, enhancing empathy, emotional resilience, and fear extinction, makes it well-suited to trauma work [52]. In phase 3 clinical trials, patients undergoing MDMA-assisted therapy exhibited significant improvements, with many no longer meeting diagnostic criteria for PTSD after the treatment protocol [53].

Importantly, these results were maintained at follow-up, suggesting durable therapeutic effects. Psychedelics have also shown efficacy in treating various forms of anxiety, particularly in existential contexts [54]. Studies involving terminally ill patients with cancer have found that psilocybin can dramatically reduce end-of-life anxiety and existential distress [55]. Participants often report profound shifts in perspective, including a greater sense of peace, interconnectedness, and acceptance of mortality. These changes are attributed not merely to neurochemical alterations but to the subjective content of the psychedelic experience, which frequently includes insights related to life, death, and meaning [56].

In the realm of addiction treatment, psychedelics have demonstrated notable potential [57]. Psilocybin has been studied for smoking cessation and alcohol use disorder, with encouraging outcomes [58]. Mechanistically, psychedelics are thought to disrupt compulsive patterns of thought and behavior, enhance motivation for change, and catalyze self-reflection. Ayahuasca and ibogaine, though less studied in Western contexts, have long been used in Indigenous and therapeutic settings for the treatment of substance dependence, offering further avenues for exploration [59].

### 4.1. Combined Therapy Models and Integration

A key aspect of psychedelic-assisted treatment is its reliance on a holistic therapeutic model that integrates pharmacological intervention with guided psychological support [60]. This typically involves a structured protocol composed of three phases: preparatory sessions before the psychedelic experience, a supervised dosing session, and a series of post-session integration meetings [61]. The preparatory phase is designed to establish rapport between the therapist and the patient, set clear therapeutic intentions, and explore potential psychological themes or concerns that may arise. This phase helps orient the participant to the importance of mindset (“set”) and physical and social environment (“setting”), which are both critical determinants of the therapeutic outcome [62].

During the psychedelic session itself, patients are often encouraged to adopt a receptive, inward-focused attitude, typically while lying down with eyeshades and listening to a curated music playlist [63]. The therapist or guide maintains a supportive, non-intrusive presence, providing emotional grounding and safety [64]. Unlike traditional talk therapy, the dosing session emphasizes internal processing, allowing the psychedelic compound to facilitate spontaneous emotional, cognitive, or somatic experiences that may emerge. These experiences can include revisiting traumatic memories, encountering symbolic imagery, confronting existential concerns, or experiencing a sense of unity or transcendence.

The final phase, post-session integration, is perhaps the most crucial for long-term therapeutic success [42]. Integration involves revisiting the experience in a coherent, structured way, using psychotherapy, journaling, body work, or creative expression to process the insights gained [65]. The goal is to translate fleeting realizations into sustainable behavioral and emotional change. Without proper integration, the psychedelic experience risks becoming disconnected from the patient’s everyday life, potentially leaving them disoriented or emotionally exposed, Gorman suggests. Research increasingly supports the notion that the quality and duration of integration work are strong predictors of long-term outcomes [66]. As such, psychedelic therapy is not a one-time pharmacological intervention but an extended therapeutic process that requires careful preparation, presence, and post-experience reflection.

### 4.2. Challenges and Considerations

While clinical results from psychedelic research have been promising, the translation of these therapies into standard psychiatric practice involves a series of logistical, methodological, and ethical challenges. One of the most pressing issues is the standardization of treatment protocols [67]. Given the profound subjectivity of psychedelic experiences, no two sessions are alike. This variability complicates the task of creating universal guidelines for dosage, session structure, and therapeutic technique [61]. Unlike psychopharmacological treatments that act more uniformly across populations, psychedelics demand a flexible, individualized approach. However, such flexibility also presents challenges for clinical reproducibility and regulatory approval, which typically require standardized protocols and measurable outcomes [68].

Another significant barrier is the shortage of adequately trained professionals. The role of a psychedelic therapist differs substantially from traditional psychotherapists [69]. Facilitating non-ordinary states of consciousness requires specialized training in trauma-informed care, transpersonal psychology, somatic awareness, and crisis management [70]. Currently, few accredited programs exist to meet this demand, and many therapists are unfamiliar or uncomfortable with the altered states induced by psychedelics. This highlights the urgent need for formal certification pathways, clinical supervision, and ethical oversight specific to this modality.

In addition to clinical and training limitations, accessibility is an increasingly prominent concern [71]. Psychedelic therapy is time-intensive, typically involving multiple preparatory and integration sessions as well as a full-day dosing experience [72]. This format may be prohibitively expensive, especially in private clinical settings [73]. As health systems begin to consider reimbursement models, questions of insurance coverage, equity, and health justice arise [74].

Finally, there are ongoing concerns around the potential for overmedicalization or commodification of psychedelic substances, especially as commercial interest grows [75]. Without careful ethical consideration and inclusive policy frameworks, the rollout of psychedelic therapy risks becoming a service accessible only to privileged populations, potentially reinforcing the disparities it aims to address [76].

## 5. Pharmacology and Safety Considerations

The therapeutic promise of psychedelics is intimately tied to their unique pharmacological properties, which govern their effects, duration, and safety profiles. Understanding these factors is critical for clinicians, researchers, and policymakers to develop responsible and effective treatment protocols. Classic psychedelics encompass a diverse range of chemical compounds that interact primarily with serotonin receptor systems but differ widely in their pharmacokinetics, dosage parameters, and potential adverse effects [77]. This section explores the pharmacological mechanisms of major psychedelics, their safety considerations, and the importance of dosage and administration context in minimizing risks.

### 5.1. Pharmacological Mechanisms

Classic psychedelics, such as lysergic acid diethylamide (LSD), psilocybin, and dimethyltryptamine (DMT), are primarily serotonin receptor agonists, particularly at the 5-HT2A receptor subtype [78]. Activation of these receptors leads to widespread changes in cortical activity, neural connectivity, and sensory processing, producing the characteristic alterations in perception, cognition, and mood [79].

Psilocybin is rapidly converted into psilocin after ingestion [80], which then crosses the blood–brain barrier and stimulates 5-HT2A receptors [81]. This receptor activation is thought to disrupt normal patterns of network activity such as the default mode network (DMN) [82]. LSD similarly acts as a potent 5-HT2A agonist but also binds to dopaminergic and adrenergic receptors, contributing to its longer duration and more complex psychopharmacological profile [83]. MDMA, often classified as an entactogen rather than a classic psychedelic, primarily increases the release of serotonin, dopamine, and norepinephrine, fostering empathy and emotional openness rather than vivid hallucinations [84]. Ketamine, a dissociative anesthetic used off-label as a rapid-acting antidepressant, acts as an NMDA receptor antagonist, modulating glutamate transmission and promoting synaptic plasticity through distinct mechanisms from serotonergic psychedelics [85].

### 5.2. Pharmacokinetics

Pharmacokinetic properties, including absorption, distribution, metabolism, and elimination, influence onset, duration, and intensity of psychedelic effects. Psilocybin is rapidly metabolized to psilocin by alkaline phosphatases in the gut and liver, with peak plasma concentrations occurring within 60 to 90 min [86]. LSD is well absorbed orally, with peak effects emerging after 1 to 2 h and a half-life of approximately 3 h [87].

MDMA’s pharmacokinetics are more complex due to its dual role as a releaser and reuptake inhibitor of monoamines [88]. Its metabolism involves liver enzymes such as CYP2D6, with notable individual variability affecting duration and intensity [89]. Ketamine’s rapid metabolism through the cytochrome P450 system produces active metabolites like norketamine, which contribute to its antidepressant effects [90].

### 5.3. Safety and Adverse Effects

While psychedelics are generally considered physiologically safe when administered in controlled environments, their potent psychoactive effects necessitate careful consideration of psychological safety. Classic psychedelics have low toxicity profiles and minimal addictive potential [91]. However, acute adverse effects may include anxiety, panic, paranoia, confusion, nausea, and transient increases in blood pressure and heart rate [92].

Psychological risks are primarily linked to “challenging experiences” or “bad trips,” which can provoke distress, disorientation, or exacerbation of underlying psychiatric conditions, especially in individuals with psychotic disorders or a family history of schizophrenia [93]. Thus, thorough screening and exclusion criteria are critical in clinical trials and therapeutic settings, Schlag highlights. MDMA carries additional concerns related to hyperthermia, dehydration, and potential serotonergic neurotoxicity, particularly when used recreationally at high doses or in uncontrolled settings [94]. Clinical protocols carefully monitor vital signs and control dosing to mitigate these risks [95]. Ketamine’s safety profile includes potential dissociative symptoms, transient blood pressure elevations, and the risk of abuse or dependence with frequent use [96]. Its use in clinical settings requires medical supervision and attention to dosing intervals.

### 5.4. Set and Setting: Crucial Determinants of Safety

Beyond pharmacology, the context of administration, often termed “set and setting”, plays a decisive role in safety and therapeutic outcomes [97]. “Set” refers to the individual’s mindset, expectations, and psychological preparedness, while “setting” encompasses the physical, social, and cultural environment [98]. Clinical studies emphasize that well-prepared participants, supportive therapeutic environments, and trained facilitators greatly reduce the risk of adverse psychological reactions, Haijen explains. Conversely, uncontrolled or unsupervised use increases the likelihood of challenging experiences and potential harm.

### 5.5. Drug Interactions and Contraindications

Psychedelics may interact with other medications, most notably selective serotonin reuptake inhibitors (SSRIs) and monoamine oxidase inhibitors (MAOIs), potentially altering efficacy or increasing side effect risk [99]. For instance, MAOIs potentiate DMT effects in ayahuasca but also raise risks of hypertensive crises when combined with certain foods or drugs [77]. Contraindications for psychedelic therapy typically include a personal or family history of psychosis, bipolar disorder, or severe cardiovascular disease [100]. These exclusions safeguard against exacerbation of symptoms or unpredictable physiological stress, Frescska highlights.

### 5.6. Emerging Safety Protocols and Training

As clinical research expands, formalized safety guidelines and training programs for psychedelic-assisted therapy are being developed [101]. These include standardized screening tools, monitoring protocols during sessions, and integration practices to ensure psychological support post-treatment. Professional organizations and regulatory bodies are also formulating best practices to minimize risks and maximize benefits as psychedelics transition from experimental to approved therapeutic tools [102].

## 6. Anthropological and Cultural Dimensions

Psychedelic substances have been intimately woven into the fabric of human culture for thousands of years, playing vital roles in spiritual, social, and healing traditions around the world [103]. The contemporary revival of interest in psychedelics within Western medicine and science stands in contrast to the rich, continuous histories of Indigenous peoples who have preserved these practices over millennia [104]. A thorough and nuanced understanding of psychedelics today requires situating these compounds within their anthropological and cultural contexts, recognizing the meanings, rituals, and communal identities they have historically shaped.

Across many Indigenous societies, psychedelics have been employed as sacraments, healing agents, and mediators of spiritual connection [105]. In the Amazon basin, for example, the ayahuasca brew, composed of the *Banisteriopsis caapi* vine combined with leaves containing the psychoactive compound DMT, has been used for centuries in shamanic ceremonies, Williams states. These rituals, guided by experienced healers or shamans, serve to diagnose illnesses, restore harmony within individuals and their communities, and facilitate communication with the spiritual world [106]. The ceremonial context, complete with chanting, drumming, and group participation, is critical to the meaning and effectiveness of the experience [107]. It is not simply the pharmacological properties of the brew that matter but also the collective framework that shapes the journey.

Similarly, peyote, a cactus containing mescaline, holds a sacred status within the Native American Church [108]. Used in religious ceremonies that aim to foster spiritual insight and moral guidance, peyote is protected under legal exemptions in some countries, reflecting recognition of its deep cultural and religious significance. Its use within this context is highly structured and governed by communal values, serving as a living link between participants and their ancestors.

Anthropological studies highlight that psychedelic use cannot be reduced to a simple chemical interaction; rather, it is fundamentally shaped by the cultural environment and belief systems in which it occurs [109]. The significance attributed to the experience, the expectations established by ritual, and the presence of knowledgeable guides or elders profoundly influence the outcomes. This understanding forms the foundation for the contemporary concept of “set and setting,” which emphasizes psychological readiness and environmental context as essential to the safety and therapeutic efficacy of psychedelics [110].

In Western contexts, the resurgence of psychedelics has often drawn heavily on Indigenous knowledge and ceremonial practices [105]. However, this borrowing has sparked important ethical debates around cultural appropriation, intellectual property, and the commodification of traditional spiritual practices. While Indigenous knowledge has undeniably enriched modern therapeutic models, questions remain about how to respect and protect the rights of source communities, ensure fair benefit-sharing, and avoid erasing the cultural origins of these substances [111].

The globalization of psychedelic use has also led to the emergence of hybrid cultural forms [112]. Western participants frequently travel to South America to take part in ayahuasca ceremonies or attend retreats that blend traditional Indigenous rituals with modern psychotherapy [113]. These cross-cultural exchanges create new practices that combine elements from multiple traditions, often reflecting a globalized spirituality that resonates with contemporary seekers. At the same time, they raise complex issues of authenticity and cultural sensitivity, as well as concerns about the sustainability and regulation of these ceremonies.

Importantly, anthropological research cautions against assuming that psychedelic experiences are universally consistent [114]. Instead, cultural frameworks significantly shape the phenomenology of the experience itself, how visions are interpreted, what emotions are evoked, and the meanings assigned to insights. Thus, the subjective effects of psychedelics are not purely pharmacological but deeply intertwined with the user’s cultural background, language, and worldview.

The role of psychedelics in traditional societies often extends beyond the individual, emphasizing collective healing, social cohesion, and the reinforcement of shared values [110]. Ritual use frequently functions to maintain community harmony, address social tensions, and link participants to ancestral wisdom. This contrasts with many Western therapeutic models that prioritize individual psychological outcomes, suggesting that integrating communal and cultural dimensions could enrich modern psychedelic practices [115].

As contemporary research continues to develop, it is increasingly clear that the cultural and anthropological dimensions of psychedelics offer invaluable insights into their potential uses and limitations [116]. Respecting these perspectives challenges reductionist approaches and invites a more holistic view that incorporates spirituality, ritual, and relationality. Moreover, engaging with Indigenous communities as partners rather than mere sources of knowledge is essential for ethical progress in the field, Batchelder states.

To better understand the global landscape of psychedelic use, Table 1 provides a comparative overview of selected countries with respect to the therapeutic legality of psychedelic substances, their historical usage, and the cultural contexts in which they have traditionally been employed [117,118]. This overview highlights both the resurgence of interest in modern clinical settings and the deep-rooted cultural practices that have shaped the historical significance of these compounds across diverse societies.

## 7. Philosophical and Existential Implications

The use of psychedelics opens profound philosophical and existential questions that challenge conventional understandings of consciousness, selfhood, and reality [119]. Beyond their therapeutic and neurobiological effects, psychedelics have historically been regarded as gateways to altered states of consciousness that can transform one’s fundamental sense of identity, meaning, and purpose [30]. These substances invite inquiry into the nature of subjective experience, the construction of the self, and the ways humans find significance in existence.

One of the central philosophical questions psychedelics raise concerns the nature of consciousness itself [10]. Psychedelic experiences often involve dramatic alterations in perception, cognition, and awareness, including vivid visual phenomena, synesthesia, and a dissolution of boundaries between self and environment [120]. Such states challenge Cartesian dualism, the notion that mind and body are distinct entities, and instead support monistic or non-dual perspectives that emphasize interconnectedness and the fluidity of mental phenomena [121]. Philosophers and cognitive scientists have debated whether these experiences reveal hidden aspects of consciousness or simply result from disrupted neural processes [122]. Regardless, they highlight the profound plasticity and contextuality of conscious experience.

Closely related is the phenomenon of ego dissolution, frequently reported during high-dose psychedelic sessions [123]. Users describe a loss of the usual sense of self as a bounded, separate entity, replaced by a feeling of unity with the cosmos or an expansive awareness that transcends personal identity, Mason highlights. This experience challenges foundational ideas about the self as a continuous, autonomous subject. Philosophically, it raises questions about the nature and persistence of personal identity, inviting interpretations ranging from mystical insights into universal consciousness to neuroscientific models of transient self-representation [124]. Ego dissolution also bears significant existential implications. The experience of self-transcendence can provoke profound shifts in one’s relationship to mortality, suffering, and purpose [125]. Many individuals report a newfound appreciation for life’s interconnectedness and impermanence, leading to changes in values, priorities, and behavior. From this perspective, psychedelics can be seen as tools that facilitate existential exploration, helping users confront anxieties about death and meaninglessness with a sense of acceptance and awe [126].

Furthermore, psychedelic experiences often generate encounters with what users interpret as spiritual or mystical realities [127]. These may include visions of divine beings, encounters with archetypal symbols, or feelings of profound sacredness. Such phenomena align with perennial philosophical questions about the transcendent and the numinous, experiences that evoke a sense of awe and reverence beyond ordinary understanding [128]. These mystical dimensions of psychedelics have been linked to positive therapeutic outcomes, suggesting that meaning-making processes rooted in spirituality can be integral to psychological healing, Samiento writes.

Philosophically, these spiritual experiences raise challenging questions about the ontology of mystical phenomena [129]. Are these experiences mere hallucinations produced by altered brain chemistry, or do they access some deeper metaphysical truths? Scholars remain divided, with some advocating for a materialist reductionism that explains mystical states as brain states, while others propose that psychedelics may reveal aspects of reality normally inaccessible to ordinary consciousness [130]. This debate touches on broader issues in the philosophy of mind and metaphysics, including the limits of scientific explanation and the validity of subjective experience as a source of knowledge.

In addition to challenging concepts of self and consciousness, psychedelics also influence moral and ethical frameworks. The profound empathy, connectedness, and dissolution of ego boundaries reported by many users can foster a heightened sense of compassion and ethical responsibility toward others and the environment [131]. This shift in perspective is sometimes described as a “moral awakening,” where users feel motivated to live more authentically, altruistically, and in harmony with the natural world [132]. Such transformations echo philosophical traditions that emphasize the interconnectedness of beings and the ethical imperative to care for others.

The existential reorientation catalyzed by psychedelics can also challenge dominant cultural narratives around individualism, materialism, and control. Many users report a diminished fixation on consumerism, competition, and ego-driven pursuits, replaced by values emphasizing connection, creativity, and presence [133]. This shift suggests that psychedelics might play a role in broader societal change by encouraging alternative ways of understanding human flourishing and well-being.

However, these profound experiences and insights are not universally transformative or positive. Psychedelics can also provoke existential crises, anxiety, or disorientation, particularly when taken without adequate preparation or support [134]. Byock suggests that the destabilization of the self-concept can be deeply unsettling, raising philosophical questions about the stability and coherence of identity. The challenge for therapeutic and philosophical inquiry lies in integrating these experiences in a way that supports lasting psychological growth and ethical engagement.

Finally, the resurgence of psychedelic research invites a reevaluation of the role altered states of consciousness play in human cognition and culture [135]. Philosophers have long pondered the nature of imagination, creativity, and insight. Psychedelics appear to expand the boundaries of ordinary cognition, enabling novel connections, perspectives, and ways of understanding [136]. This suggests that altered states may have evolutionary significance, contributing to human adaptability and cultural innovation.

## 8. Medical Uses Beyond Psychiatry

The therapeutic promise of psychedelics has largely focused on psychiatric disorders such as depression, PTSD, anxiety, and addiction [137]. However, emerging evidence suggests that their potential medical applications extend beyond the domain of mental health [138]. Psychedelics may offer novel interventions for chronic pain [139], palliative care [140], and somatic conditions where conventional treatments have limited efficacy [141]. These expanded uses are supported by both mechanistic insights from neuroscience and a growing number of clinical observations, indicating that psychedelics may exert complex effects on perception, emotion, and physiological systems that intersect with physical health and healing [142].

One of the most promising frontiers in non-psychiatric psychedelic medicine is in the treatment of chronic pain [143]. Pain is not solely a sensory phenomenon but involves affective, cognitive, and emotional dimensions [144]. Psychedelics, particularly classical serotonergic compounds like psilocybin and LSD, appear to influence pain perception through both psychological and neurobiological mechanisms [145].

Beyond these neurobiological mechanisms, the subjective experience of psychedelics can shift patients’ relationship to their pain [146]. In qualitative reports, individuals describe experiencing their pain from a new perspective, often with increased acceptance and reduced emotional reactivity. Bornemann highlights that this is consistent with observations in mindfulness-based pain management, where changing the cognitive appraisal of pain can significantly improve quality of life. By disrupting habitual thought patterns and promoting cognitive flexibility, psychedelics may reduce the suffering component of pain, even if the physical sensation remains.

A particularly compelling area of research involves the use of psychedelics in palliative care and end-of-life settings [56]. Patients facing terminal illness often struggle with existential distress, fear of death, and a loss of meaning, Federico explains. Traditional pharmacological treatments for such distress, such as benzodiazepines or antidepressants, are often inadequate and may dull consciousness without addressing the deeper psychological and spiritual dimensions of dying [147]. Psychedelic-assisted therapy, by contrast, has shown remarkable promise in alleviating anxiety and depression in terminally ill patients, often after just a single guided session.

Studies using psilocybin for cancer-related psychological distress have demonstrated rapid and sustained improvements in mood, anxiety, and existential well-being [148]. Participants frequently report a decreased fear of death, greater emotional acceptance, and a renewed sense of connection to life, loved ones, and the universe [149]. These effects are not simply byproducts of mood elevation but often emerge from what participants describe as deeply meaningful, even mystical experiences. Such outcomes suggest that psychedelics may uniquely address the spiritual and existential needs of patients at the end of life, offering a level of psychological and emotional relief that is rarely achieved by conventional means.

In terms of clinical application, patient selection, dosing strategies, and integration support must all be adapted to the specific medical context. For instance, the emotional and existential needs of a terminal cancer patient differ significantly from those of a person with chronic back pain. Tailoring the therapeutic approach to fit the patient’s medical, psychological, and existential profile will be key to maximizing benefits and minimizing risks.

## 9. Ethical and Methodological Challenges in Psychedelic Research

The recent resurgence of psychedelic research has reinvigorated scientific and medical discourse, but it has also exposed a complex landscape of ethical and methodological challenges. Psychedelics, unlike many conventional pharmaceuticals, involve deeply subjective, often unpredictable experiences that are profoundly shaped by context, expectation, and interpersonal dynamics [150]. As such, designing robust, ethical studies that meet the standards of modern scientific inquiry while honoring the complexity of these substances presents a unique set of difficulties. Addressing these challenges is critical for ensuring the safe, effective, and responsible development of psychedelic-assisted therapies.

One of the most immediate ethical concerns in psychedelic research involves participant safety and informed consent. Psychedelic substances, especially psilocybin, LSD, and DMT, can induce powerful alterations in consciousness, including emotional catharsis, perceptual distortions, and ego dissolution [151]. While these effects may contribute to therapeutic breakthroughs, they can also be psychologically destabilizing or overwhelming, particularly for individuals with a history of trauma or latent mental health vulnerabilities. Ensuring that participants are adequately screened, informed of potential risks, and supported throughout the experience is essential. Informed consent must go beyond a standard legal disclosure to include detailed preparation about the nature of the psychedelic experience, the importance of “set and setting,” and the role of the guide or therapist [152].

The therapeutic environment itself presents another layer of ethical complexity [153]. Unlike conventional drug trials, which focus on standardized pharmacological testing, psychedelic therapy is not a clinical trial but a therapeutic process that relies on the interpersonal relationship between the participant and the facilitator [61]. This places significant responsibility on therapists, who must balance clinical neutrality with the emotionally intimate role often required to support participants during vulnerable states. Boundaries can become blurred, particularly when participants experience transference or come to regard the therapist as a spiritual or parental figure. Ethical guidelines must therefore emphasize therapist training, supervision, and the prevention of misconduct [61]. Historical instances of boundary violations in psychedelic therapy underscore the urgency of establishing professional standards and accountability mechanisms [154].

Methodologically, psychedelic research must contend with the challenge of placebo control [155]. The effects of psychedelics are typically unmistakable, making it exceedingly difficult to blind participants and researchers to treatment conditions, Wen highlights. This lack of blinding introduces expectancy effects and biases that can confound results. Some studies have attempted to use active placebos, such as low-dose psychedelics or other mildly psychoactive substances like niacin, but these often fail to mimic the intensity of the psychedelic experience [156]. Consequently, researchers must interpret findings with caution and consider alternative designs that account for non-specific factors, such as participant expectations and the therapeutic alliance.

Moreover, the question of what constitutes an appropriate control group in psychedelic studies remains unresolved. Unlike conventional medications that aim to produce predictable, dose-dependent outcomes, psychedelics operate in a context-dependent manner, where psychological, cultural, and environmental variables strongly influence results [157]. This complexity makes it difficult to isolate the specific contribution of the drug from the broader therapeutic framework. As a result, researchers have called for “whole-system” or “context-inclusive” approaches that evaluate not only the substance but also the setting, preparation, and integration processes as integral components of the treatment.

Another significant methodological challenge involves the integration of subjective reports into scientific analysis. Psychedelic experiences are inherently personal and often described in metaphorical or spiritual terms, which resist straightforward quantification [158]. While standardized psychometric instruments such as the Mystical Experience Questionnaire [159] and the Ego Dissolution Inventory [125] provide some structure for data collection, they cannot fully capture the richness and variability of individual experiences. This tension between subjective depth and empirical rigor calls for mixed-methods approaches that combine quantitative measures with qualitative interviews, narrative analysis, and phenomenological inquiry [160].

The growing commercialization of psychedelics introduces ethical dilemmas that must be addressed at both institutional and policy levels [161]. As pharmaceutical companies, retreat centers, and venture capitalists enter the psychedelic space, there is increasing pressure to prioritize profitability over patient welfare, scientific integrity, and equitable access [162]. Patenting of psychedelic compounds, delivery methods, or therapeutic protocols raises concerns about intellectual property monopolies that could limit public access and stifle collaborative innovation. These dynamics risk commodifying substances that have been used ceremonially by Indigenous cultures for centuries, often without their consent or benefit.

Finally, regulatory frameworks have not yet fully adapted to the unique demands of psychedelic research. Despite increasing evidence of safety and efficacy, psychedelics remain Schedule I substances under international conventions and in many national jurisdictions, meaning they are classified as having no accepted medical use and a high potential for abuse [163]. This classification imposes significant logistical and financial barriers for researchers, including complex licensing procedures, security requirements, and stigma within academic institutions, Nutt highlights. Reforming drug policy to reflect current scientific understanding is essential for advancing the field and enabling broader access to potentially life-changing treatments.

## 10. Conclusions

The contemporary resurgence of interest in psychedelics marks a pivotal moment in both psychological science and broader interdisciplinary thought. Once relegated to the fringes of medical and cultural discourse due to prohibition and stigma, psychedelics are now at the forefront of a paradigm shift that bridges neuroscience, psychiatry, anthropology, philosophy, and medical ethics. This renaissance is not merely a practice of mid-20th-century investigations but a data-driven and context-aware movement that seeks to responsibly integrate these potent substances into modern therapeutic and epistemological frameworks.

One of the most profound revelations from recent research is the remarkable therapeutic efficacy of psychedelics in addressing a wide range of psychiatric conditions, including treatment-resistant depression, PTSD, anxiety, and addiction. Unlike conventional pharmacotherapies that often aim to suppress symptoms, psychedelics appear to facilitate transformative psychological change by enhancing introspection, emotional processing, ego dissolution, and neuroplasticity. These therapeutic effects are often catalyzed through just one or two experiences yet yield outcomes that can last for months, a finding that challenges the chronic treatment models underpinning much of modern psychiatry.

Equally compelling are the neurobiological underpinnings of these effects. Psychedelics modulate key brain systems, most notably the serotonin 5-HT2A receptor and the default mode network, producing a temporary loosening of rigid neural patterns that may enable more flexible cognition and emotional regulation. By promoting neural connectivity and fostering conditions conducive to the reorganization of mental frameworks, these compounds serve not only as symptom relievers but as agents of psychological restructuring.

Moreover, psychedelic-assisted therapy is inherently integrative, combining pharmacological intervention with structured psychological support. This therapeutic model emphasizes the importance of “set and setting,” integration, and the relational dynamic between guide and participant, highlighting the contextual nature of healing. These elements mark a departure from traditional Western biomedical models and point toward a more holistic approach to mental health, one that recognizes the interplay between biology, emotion, meaning, and social context.

Beyond the clinic, psychedelics raise far-reaching philosophical and cultural questions. Experiences of ego dissolution, unity, and mystical insight prompt reevaluation of entrenched concepts of self, consciousness, and reality. These phenomena echo long-standing spiritual and existential inquiries found across human history and cultures, suggesting that psychedelics may have a unique role in fostering not only psychological healing but also moral reflection, creativity, and personal growth. The potential for psychedelics to catalyze shifts in values toward empathy and connectedness also implies relevance beyond individual therapy, pointing to their possible contributions to social and ecological transformation.

Anthropological perspectives further enrich this discussion by situating psychedelics within the cultural traditions from which many of them originate. Indigenous communities have long utilized these substances within ritualized frameworks aimed at healing, cohesion, and spiritual communion. Acknowledging and respecting these traditions is essential, not only for ethical integrity but also for expanding Western paradigms of medicine and healing. As psychedelic therapy enters mainstream discourse, it is imperative that it does so with cultural humility, inclusive dialogue, and mechanisms for fair benefit-sharing.

Nevertheless, the integration of psychedelics into modern health systems is not without challenges. Ethical considerations around consent, safety, and therapist conduct must be rigorously addressed. Methodological limitations, such as difficulties in achieving placebo controls or standardizing subjective experiences, complicate the evidentiary base. Furthermore, concerns about access, equity, commercialization, and overmedicalization must be met with thoughtful policy and inclusive frameworks to prevent the marginalization of vulnerable populations and the co-optation of Indigenous knowledge.

Despite these complexities, the momentum behind psychedelic research is undeniable. With growing support from academic institutions, regulatory bodies, and the public, there is an unprecedented opportunity to reimagine mental health care. This reimagining calls for a delicate balance between scientific rigor and openness to subjective, spiritual, and cultural dimensions of healing. It demands innovation in research methodologies, investment in training and ethical guidelines, and a commitment to equity and justice.

Psychedelics invite us to expand our definitions not only of therapy and medicine but also of consciousness, suffering, and what it means to be whole. Their reemergence challenges reductionist models and offers a more expansive, integrative vision of human well-being. As science, culture, and policy converge around this promising frontier, the question is no longer whether psychedelics have a role to play in contemporary inquiry but how we will responsibly shape and share that role for generations to come.

## Figures and Tables

**Table 1:** Legality, historical use, and cultural context of psychedelics [117,118].

Country	Psychedelics	Legality for Therapy	Historically Used	Cultural/Traditional Use
USA	Psilocybin, MDMA, ketamine	Partially (state-level or trial-based)	No	Modern clinical trials; underground therapy; state decriminalization (e.g., Oregon, Colorado)
Canada	Psilocybin, MDMA	Yes (limited exemptions for palliative and treatment-resistant cases)	No	Legal exemptions for end-of-life therapy; growing clinical research
Brazil	Ayahuasca	Yes (legal for religious use)	Yes	Used in syncretic religions (Santo Daime, União do Vegetal)
Peru	Ayahuasca, San Pedro	Yes (in traditional and spiritual settings)	Yes	Integral to Amazonian and Andean shamanic healing ceremonies
Mexico	Psilocybin (mushrooms)	No (religious/Indigenous use tolerated)	Yes	Sacred mushroom rituals (veladas) among Mazatec and other Indigenous groups
Netherlands	Psilocybin truffles	Yes (truffles only, not mushrooms)	No	Legal truffle sales; therapeutic retreats and guided sessions
Australia	Psilocybin, MDMA	Yes (approved for PTSD and depression as of 2023)	No	Approved under medical supervision; emerging clinical framework
India	Cannabis, datura	No	Yes	Used in spiritual rites; possible Vedic references (e.g., soma)
South Africa	Psilocybin, ayahuasca	No	Possibly	Underground use; neo-shamanic practices on the rise
Portugal	Various (decriminalized)	No (but personal use is decriminalized)	No	Decriminalized personal use; informal healing and introspective applications
Switzerland	LSD, MDMA, psilocybin, ketamine, ayahuasca	Yes (licensed therapy allowed in specific cases since 2014)	No	Clinical psychedelic therapy; psycholytic therapy history; licensed ayahuasca use in Geneva

## Data Availability

No new data were created or analyzed in this study. Data sharing is not applicable to this article.
